# Preoperative Neutrophil: Lymphocyte Ratio, Platelet: Lymphocyte Ratio, and C-Reactive Protein Levels Predictive Value in Determining the Severity of Breast Mass

**DOI:** 10.30699/IJP.2022.539042.2727

**Published:** 2022-09-02

**Authors:** Aida Alizamir, Sakineh Dehghan Azad, Azar Pirdehghan, Arash Moradi

**Affiliations:** 1 *Department of Pathology, Hamadan University of Medical Sciences, Hamadan, Iran*; 2 *Department of Community Medicine, Hamadan University of Medical Sciences, Hamadan, Iran*; 3 *Department of Medical Biotechnology, National Institute of Genetic Engineering and Biotechnology, Tehran, Iran*

**Keywords:** Biomarkers, Blood Platelets, Breast Neoplasms, C-Reactive Protein, Inflammation, Neutrophils, Lymphocytes

## Abstract

**Background & Objective:**

Female breast cancer is one of the most prevalent malignancies among women. The critical step in management of breast cancer is an accurate diagnosis. Hence, peripheral blood-based tests would be one of the most favorable and less invasive methods to study. Recent studies have investigated the inflammatory parameters such as neutrophil: lymphocyte ratio (NLR), the platelet: lymphocyte ratio (PLR), and the C-reactive protein (CRP) levels. The elevation in mentioned parameters was proposed as a key factor in cancer progression. The main goal of this study was to investigate the association of NLR, PLR, and CRP levels in patients with breast lesions.

**Methods:**

The NLR, PLR, and CRP levels were calculated from 200 female patients presenting with either benign or malignant lesions.

**Results:**

The cut-off values of NLR, PLR, and CRP were 1.24, 96, and 10.36 mg/L, respectively. A significant difference in NLR (*P*<0.001), PLR (*P*<0.001), and CRP levels (*P*<0.001) were observed between the two major studied cohorts.

**Conclusion:**

Elevated NLR, PLR, and CRP levels could predict the presence of malignancy. In addition to the low cost and properties of the mentioned methods, utilization of this data could facilitate and improve clinical decision-making for treatment.

## Introduction

Breast masses are heterogeneous in etiology, and they are divided into two main groups, including benign and malignant masses. Noncancerous breast lesions, including fibroadenoma and mammary adenosis, are commonly diagnosed in adult women. They have heterogeneous histological origins, for instance, mammary epithelium or other mammary tissues (1, 2). On the other hand, female breast cancer (BC) is top-rated cancer with high incidence and mortality (3). Therefore, understanding the biology of BC would be helpful for accurate diagnosis of BC.

Diagnosis of BC at the early stages is an essential and beneficial part of BC management (4). Earlier, cancer research only focused on the cancer cell processes. Nowadays, the importance of the tumor microenvironment (TME) has been emphasized (5). Therefore, numerous studies on the potential mechanisms of inflammation have been investigated and proposed in cancer pathogenesis (6). Undoubtedly, inflammatory cells and mediators in the TME are pivotal participants in cancer cell proliferation and progression (7) and could be responsible for treatment response (8).

Neutrophils are suggested to have critical functions in the TME. They suppress the adaptive immune response in the TME and have a potential regulatory role in tumor progression (9). Markedly, neutrophils secrete Neutrophil Extracellular Traps (NETs), web-like structures comprising DNA fibers, histones, and antimicrobial proteins. They were discovered by Brinkmann and colleagues (10) and had been represented as traps for exogenous pathogens. In addition to the pivotal role of NETs as a host defense mechanism, NETs significantly impact cancer progression and metastatic dissemination (11). 

Other main players are platelets, which contribute to hemostasis and thrombosis. The activated platelets stimulate cancer-associated inflammation by regulating hematopoietic and immune cell migration toward the tumor site. It has been demonstrated that platelets and neutrophils have interactions via their surface and secreted molecules. These interactions lead to the blood clot and NETs formation, which promote cancer cell metastasis and progression by concealing them from degradation (12).

Furthermore, investigators suggest that the systemic inflammatory response could be beneficial in stratifying cancer patients. Some studies have demonstrated a significantly elevated plasma concentration of C-Reactive Protein (CRP) in response to inflammation, tissue damage (13), and numerous cancer types (14). Due to hypoxia and necrosis in cancer, CRP levels will arise as a nonspecific inflammatory response. Interestingly, studies showed elevated CRP levels are associated with impaired cell-mediated immunity and activation of the innate immune system (15). Thus, studying the systemic inflammatory response and its interaction with the immune system is beneficial for understanding the cancer biology.

Intriguingly, relative hematopoietic changes, such as neutrophil: lymphocyte ratio (NLR), platelet: lymphocyte ratio (PLR), and inflammatory response CRP levels alteration, have recently been recognized as poor prognostic indicators in various cancers (13, 16-18). Neutrophils, platelets, and lymphocytes significantly influence tumor-related inflammation and immunology (19), and CRP could reflect an immunosuppressive microenvironment (20). Therefore, their levels could be considered promising biomarkers.

Consequently, the NLR, PLR, and CRP levels in patients with breast mass comprised of benign and malignant lesions were investigated in this study. Also, the evaluated NLR, PLR, and CRP levels were investigated to whether they could be considered potential breast cancer risk factors.

## Material and Methods

**Design of Study**

This cross-sectional study was conducted from 2017 to 2018 in the Fatemiyeh Hospital of Hamadan, Iran, under the declaration of Helsinki; also, all participants signed the written and informed consent form. The participants in this study comprised women with a breast mass. Individuals with anemia, infections, specific blood diseases, inflammation, or autoimmune diseases were excluded from the study. Before surgery, none of the patients had received any other treatment, such as radiotherapy or chemotherapy. 

Before any treatment, blood samples were obtained in VACUETTE® tube 2 mL K2EDTA and VACUETTE® TUBE 4 mL CAT Serum Clot Activator. Blood-based tests, including measurement of CRP level in the patient's serum and complete blood count (CBC) from whole blood. Afterward, the NLR (Absolute Neutrophil Count / Absolute Lymphocyte Count) and PLR (Absolute Platelet Count / Absolute Lymphocyte Count) were calculated on CBC absolute results, and CRP levels were evaluated by the quantitative test. 

For histological assays, from each resected lesion, 4μm sections were cut and stained with hematoxylin and eosin for classifying the resected tissues according to the World Health Organization (WHO) criteria. Also, the Paraffin-embedded block of patients diagnosed with breast carcinoma was utilized for immunohistochemical (IHC) assay to assess ER/RP and HER2 expression.

**Statistical Analyses**

The optimal cut-off values for NLR, PLR, and CRP levels were determined by the Receiver‐operating characteristic (ROC) analysis. 

The statistical significance of differences between groups was determined using Student's t-test. Also, P-value<0.05 was considered statistically significant. 

Finally, results were expressed as means ± standard deviation (SD), and all statistical analyses were performed using GraphPad Prism 9.0.0 (GraphPad Software, Inc) software.

## Results

The patient's mean age was 42.39 ± 11.83 years. About 51.5% of participants had a benign mass, including cysts and fibroadenomas. In comparison, 48.5% of patients had malignant lesions. In addition, 57.4% of patients with malignant tumors had vascular invasion, while 8.5% had metastasis. Besides, 50% of malignant tumors had invasive ductal carcinoma phenotype (Table 1).

The ROC was used to choose the most appropriate cut-off for the NLR, PLR, and CRP levels to distinguish patients with benign mass from malignant tumors. The cut-off obtained for NLR was 1.24 (sensitivity 73.79%, specificity 80.85%, and AUC= 0.8465), for PLR was 96 (sensitivity 85.44%, specificity 98.94%, and AUC= 0.9985), and for CRP levels were 10.36 mg/L (sensitivity 100%, specificity 98.94%, and AUC= 1).

Importantly, the results have demonstrated that significant relations have been between the NLR and malignancy (*P*<0.001), vascular invasion (*P*=0.002), and metastasis (*P*=0.002). Also, the PLR had only a significant correlation with malignancy (*P*<0.001). Noteworthy, a significantly (*P*<0.001) higher CRP level was observed in patients with malignant tumors compared to patients with benign masses (Table 2).

Moreover, the NLR, PLR, and CRP levels were evaluated regarding the estrogen receptor (ER), progesterone receptor (PR), and human epidermal growth factor receptor 2 (HER2) expression. The results have demonstrated significant differences between NLR of ER^+^ (*P*=0.03) and PR^+^ (*P*=0.03) compared to ER^-^ and PR^-^, respectively. Also, the HER2^+^ group CRP levels were significantly (*P*=0.001) higher than HER2^-^ group (Table 2).

**Table 1:** Demographics and pathogenic features of patients with breast masses

Parameters		
Total number of patients		200
Mean age (year)		42.39±11.83
Age at menarche (year)		14.20±1.08
Family history (N)	Yes	53
	No	147
Age at first full-time pregnancy (year)		21.47±2.43
Body mass index (kg/m^2^)		21.55±2.49
Smoking history (N)	Yes	35
	No	165
Histology type (N)	Malignant	94
	Benign	103
Pathology type (N)	Invasive ductal carcinoma	47
	Invasive lobular carcinoma	9
	Ductal carcinoma in situ	8
	Lobular carcinoma in situ	1
	Mixed	13
	Other	16
Grade (N)	1	35
	2	37
	3	22
Stage (N)	0	5
	1	54
	2	26
	3	8
	4	1

**Table 2:** The mean ± standard deviation of NLR, PLR, and CRP was evaluated among the multiple groups

Factor (N)		NLR	PLR	CRP (mg/L)
Malignancy	Benign (94)	1.009±0.29	44.7±17	4.23±1.16
	Malignant (103)	1.44±0.31	127±33	16.68±2.67
	P-value	<0.001 *	<0.001 *	<0.001 *
	Cut-off	1.24	96	10.36
Vascular invasion	Yes (54)	1.49±0.31	135±37	16.76±3.510
	No (40)	1.25±0.26	118±23	15.89±2.85
	P-value	0.002 *	0.550	0.320
Metastasis	Yes (6)	1.98±0.28	173±24	20.71±2.371
	No (88)	1.57±0.27	124±31	15.43±3.30
	P-value	0.002 *	0.550	<0.001 *
ER	Pos (70)	1.38±0.30	128±27	16.23±2.01
	Neg (24)	1.60±0.40	133±22	16.57±1.61
	P-value	0.030 *	0.36	0.22
PR	Pos (70)	1.38±0.30	127±28	16.23±2.01
	Neg (24)	1.60±0.40	133±22	16.57±1.61
	P-value	0.030 *	0.36	0.22
HER	Pos (17)	1.53±0.18	129±26	18.75±2.45
	Neg (77)	1.38±0.30	124±25	16.54±1.90
	P-value	0.24	0.39	0.001 *

Estrogen receptor (ER), Progesterone receptor (PR), Human epidermal growth factor receptor 2 (HER2), Positive (Pos), Negative (Neg), neutrophil: lymphocyte ratio (NLR), platelet: lymphocyte ratio (PLR), C-reactive protein (CRP), and * means that there is a significant difference between groups.

## Discussion

The present study investigated neutrophil: lymphocyte ratio, platelet: lymphocyte ratio, and CRP levels based on the importance of their pivotal predictive applicability. Results have demonstrated that the group's mean of NLR, PLR, and CRP levels with malignant tumors was significantly (*P*<0.001) higher than patients with benign masses. The optimal cut-off values of NLR, PLR, and CRP were 1.24, 96, and 10.36 mg/L, respectively. This study has confirmed that NLR, PLR, and CRP levels were changed in malignant breast lesions and proposed them as predictive and/or prognostic factors (21-23).

 In addition, significant differences were observed among the NLR of ER (*P*=0.02) and PR (*P*=0.02) subtypes, but there were no significant differences in NLR within the HER2 subtypes. However, in contrast to this study's results, one metanalysis showed the prognostic value of NLR between HER2-positive and triple-Negative Breast Cancer (24). On the other hand, significant differences in CRP levels were obtained between the HER2^+^ and HER2^- ^subtypes (*P*<0.001). Ultimately, there were no significant associations between PLR and receptor status. 

Conventional screening of BC involves different types of breast imaging, such as computed tomography scan, magnetic resonance imaging, and mammography X-ray examination (25). However, they remained costly despite their widespread use. Thus, methods with lower costs and more accessibility are urgently needed. For instance, the severity of systemic inflammatory response in cancer patients with cancer can be revealed by routine hematological tests.

Numerous studies suggested that inflammation is critical in tumor development, and progression (6, 26, 27). Interestingly, the link between chronic inflammation and cancer appears reciprocal. Inflammation can promote tumor development and progression. Correspondingly, tumor development and progression can also stimulate inflammation (28). The TME is largely organized by inflammatory cells (29), specifically neutrophils, platelets, and lymphocytes contributing to tumor-related inflammation and immunology (30). The most compelling evidence is that neutrophils in the tumor microenvironment act as a pro-tumor by forming NET and promoting immunosuppression. Thus, increased neutrophil levels correlate with patients' poor outcomes (31). 

Another key point is that the activated platelets by cancer cells can stimulate venous thrombosis and NETs. Consequently, platelets protect cancer cells from shear stress and natural killer (NK) cells and facilitate cancer metastasis and progression. Also, the activated platelets regulate immune cell migration toward the tumor microenvironment, contributing to cancer-associated inflammation (12).

The neutrophil: lymphocyte ratio is superior to the parameters alone, such as neutrophil, lymphocyte, and total leukocyte count, specifically neutrophilia with a relative lymphocytopenia (32, 33) in predicting short- and long-term mortality. The NLR could be a valuable factor compared to the counts alone (34). Similarly, elevated PLR, either with thrombocythemia or lymphocytopenia, resulted in less antitumor activity and poor prognosis (35). Furthermore, the measurement of the CRP has been proved to have prognostic value in numerous types of cancer, for instance, breast cancer (36). BC patients' overall survival is inversely correlated with elevated NLR and PLR (37, 38). Also, Takeuchi *et al.* proposed that CRP levels and PLR are associated with poor prognoses in patients with BC (39).

The results of this study conveyed that elevated NLR, PLR, and CRP levels could indicate the presence of malignancy. Besides, the altered frequency of immune cells is aroused from tumor activity (40). Thus, these cells are promising targets for further investigations and targeted therapy. Developing and employing NLR, PLR, and CRP levels as biomarkers for BC may improve clinical decision-making.

Finally, there were some limitations in the present study. First, the results were obtained from a single institution using a relatively small number of subjects. Small sample sizes limit generalizability in heterogeneous diseases such as breast cancer and restrict the number of variables in a multivariate analysis. Another issue, the follow-up data of the patients with breast cancer were not available for further prospective analysis.

## Conclusion

Irrespective of the limitations mentioned above, the obtained data demonstrated that elevated NLR, PLR, and CRP levels are correlated with the presence of malignant lesions in the breasts. Harboring this information may facilitate and improve clinical decision-making for treatment. In other words, higher NLR, PLR, and CRP levels could predict the presence of malignancy. However, large-scale, and long-term studies are required to confirm the present results. 

##  Ethics Approval & Consent to Participate

This study was conducted under the declaration of Helsinki; the Ethical code was obtained from the Hamadan University of Medical Sciences with accession number IR.UMSHA.REC.1398.139. Also, all participants signed the informed consent form. 

##  Authors' Contributions

Aida Alizamir designed the study. Aida Alizamir, Sakineh Dehghan Azad, and Azar Pirdehghan contributed to the development of the methodology. Arash Moradi and Sakineh Dehghan Azad acquired the data and conducted the experiments. Aida Alizamir, Azar Pirdehghan, and Arash Moradi analyzed and interpreted data and prepared the manuscript. Aida Alizamir and Arash Moradi performed the manuscript reviews and revisions.

## Conflict of Interest

The authors declared no conflict of interest.

## Funding

The author(s) received no financial support for the research, authorship, and/or publication of this article.
